# Cleaved JAM-A - connecting cancer and vascular disease?

**DOI:** 10.18632/oncotarget.26973

**Published:** 2019-06-11

**Authors:** Cathy E. Richards, Emily J. Rutherford, Ann M. Hopkins

**Affiliations:** Department of Surgery, Royal College of Surgeons in Ireland, Ireland

**Keywords:** junctional adhesion molecule-A, clotting, HER2, inflammation

Breast cancers that overexpress the human epidermal growth factor receptor-2 (HER2) are associated with aggressive disease and poor patient prognosis [1]. Although several highly-effective HER2 targeted therapies have been developed, they are associated with a high incidence of *de novo* and acquired drug resistance in patients [1]. Therefore there remains an ongoing need to elucidate mechanisms of resistance in order to establish effective treatments in drug-resistant patients. Proteins which operate upstream of, and regulate, HER2 expression are valid pharmacological targets in this setting. In recent years a correlation between the expression of Junctional Adhesion Molecule-A (JAM-A) and HER2 in breast cancers has been reported, in addition to expressional regulation of HER2 by JAM-A [2, 3]. JAM-A is a tight junction protein with important adhesive functions in multiple physiological barriers [4], but whose overexpression has also been linked with tumor progression in glioblastoma, gastric, lung and nasopharyngeal cancers, as well as an emerging role in cancer stem cell maintenance [4, 5].

We recently demonstrated that JAM-A overexpression and its extracellular cleavage by A Disintegrin And Metalloproteinase enzymes (ADAM-10 and, to a lesser extent, ADAM-17) correlated with conditional resistance to HER2-targeted therapies in breast cellular models [6]. Importantly, analysis of a pilot study of serum samples from 20 HER2-positive breast cancer patients revealed significantly higher levels of cleaved JAM-A (cJAM-A) in the 11 patients who had developed resistance to HER2-targeted therapies.

While the precise mechanistic link between cJAM-A and clinical drug resistance remains to be elucidated, it may be the case that cJAM-A acts a novel ligand to drive invasive or other pro-tumorigenic events in breast cancer. In support of this hypothesis, we demonstrated increased invasive potential in breast cell lines exposed to recombinant cleaved JAM-A (rcJAM-A); accompanied by deeper invasion and increased positivity for the proliferation marker Ki67 in a chick embryo tumor xenograft model [6].

Cleaved soluble JAM-A may also have clinical relevance in settings outside oncology, with evidence of its elevation in the plasma of patients with angiographically-proven coronary artery disease (and in fact a direct correlation between disease score and the levels of both soluble JAM-A and the pro-inflammatory cytokine TNF-α [7]). Furthermore, although JAM-A is constitutively expressed on normal platelets, cleaved soluble JAM-A levels in the bloodstream of patients have been associated with hypertension independent of several other long-established cardiovascular risk factors including body mass index and insulin resistance [8].

The possible links between cJAM-A, cardiovascular disease and cancer are intriguing in light of the fact that both malignancy itself and various treatments (eg. chemotherapeutic agents, hormonal treatments such as Tamoxifen) or interventions (eg. central venous catheters) are recognised to increase the risk of pathological clotting. Since cancer-associated thrombosis is a major cause of morbidity and mortality, the maintenance of clotting/ bleeding homeostasis is of significant clinical relevance in cancer patient populations. One might speculate that the common denominator is inflammation, and indeed a combination of TNF-α and IFN-γ has been shown to increase the levels of cJAM-A shed from cultured endothelial cells or into the bloodstream of rodent models [9]. Interestingly, JAM-A cleavage in the endothelial models was more dependent upon ADAM17 than ADAM10 [9], whereas the reverse was the case in our breast epithelial models [6]. This highlights the importance of context-dependence, in addition to the likelihood that multiple players may contribute to the events orchestrating JAM-A cleavage in patients.

Taken together, these points suggest that JAM-A cleavage merits investigation as a biomarker or novel therapeutic target in the setting of both cancer and vascular diseases. However much remains unknown about the role of JAM-A, or its cleavage, in their inter-connectedness. For example, hypercoagubility has been reported in patients with glioblastoma brain tumors [10], and JAM-A has recently been established as an essential mediator of cancer stem cell (CSC) self-renewal in glioblastomas [5]. Could JAM-A cleavage connect those events? If so, what are the driving forces for its cleavage, and what impact would cJAM-A have on CSC abundance or function in other clinical scenarios such as haematological malignancies? Addressing these and related questions may contribute to our understanding of how CSCs drive local invasion and distant metastasis of tumors, as well as disease recurrences within patient cohorts who originally responded to therapies such as the HER2-targeted therapies. Finally, we propose that targeting JAM-A may reveal novel insights into the biological mechanisms underlying a spectrum of conditions with significant socio-economic impact.
